# Interaction of 9-Methoxyluminarine with Different G-Quadruplex Topologies: Fluorescence and Circular Dichroism Studies

**DOI:** 10.3390/ijms221910399

**Published:** 2021-09-27

**Authors:** Joanna Nowak-Karnowska, Agata Głuszyńska, Joanna Kosman, Grażyna Neunert, Anna Dembska

**Affiliations:** 1 Department of Bioanalytical Chemistry, Faculty of Chemistry, Adam Mickiewicz University, Uniwersytetu Poznańskiego 8, 61-614 Poznań, Poland; j.nowak@amu.edu.pl (J.N.-K.); kosman@amu.edu.pl (J.K.); 2 Department of Physics and Biophysics, Faculty of Food Science and Nutrition, Poznań University of Life Sciences, Wojska Polskiego 38/42, 60-637 Poznań, Poland; grazyna.neunert@up.poznan.pl

**Keywords:** G-quadruplex, *c-MYC*, 9-methoxyluminarine, fluorescence, CD spectroscopy

## Abstract

The interactions of G–quadruplexes of different topologies with highly fluorescent 9-methoxyluminarine ligand 9-MeLM were investigated by fluorescence and circular dichroism spectroscopy. The results showed that 9-methoxyluminarine was able to interact and did not destabilize any investigated molecular targets. The studied compound was selectively quenched by parallel c-*MYC* G-quadruplex DNA, whereas hybrid and antiparallel G4 topology caused only a negligible decrease in the fluorescence of the ligand. A high decrease of fluorescence of the ligand after binding with *c-MYC* G-quadruplex suggests that this molecule can be used as a selective probe for parallel G-quadruplexes.

## 1. Introduction

Guanine-rich sequences able to fold into non-canonical four-stranded nucleic acid structures under physiological conditions, called G-quadruplexes (G4s), have received particular attention in recent years due to their interesting structural features and biological functions [1]. These sequences are present in the human genome, particularly in telomeric DNA repeats [2] and key regulatory regions of the cell, such as promoters of proto-oncogenes (c-*MYC* [3,4,5,6], *BCL*-2 [7,8] and c-*KIT* [9,10,11]), and in untranslated regions of mRNA [12,13] and telomeric repeat-containing RNA (TERRA) [14,15]. There are several computational tools dedicated to searching potential G4-forming sequences in the human genome [16,17,18,19]. Intensive studies have revealed the formation of G-quadruplexes in vivo [20,21,22,23] and have provided evidence of their role in several key cellular processes, including gene transcription, chromatin epigenetics and DNA recombination. On the other hand, the linkage of G-quadruplex forming sequences with cancer diseases contributed to the rapid development of research focused on finding ligands, which can induce, stabilize or disrupt the G4 structure [24,25,26,27]. Since 2013, there has been a database containing the ligands tested against G-quadruplexes [28]. So far, only two G-quadruplex ligands, Quarfloxin (CX-3543) and CX-5461, have reached clinical trial stages against cancer [26]. Importantly, three binding modes are recognized for G-quadruplex–ligands complexes: they can stack upon the terminal G-quartet via π-π interactions between ligand and the terminal G-tetrad (external stacking); intercalation between two G-quartets; or binding to the grooves (via electrostatic interaction with the negatively charged phosphodiester backbone of the DNA) [29]. Initially it was considered that G-quadruplex binding ligands should possess structural components such as a polycyclic aromatic core, containing one or more cationic side chains (usually protonated amino groups) attached to the aromatic core [29,30]. The side chains were to contribute to the stability of G-quadruplexes by establishing electrostatic interactions with atoms in grooves and central channels [31]. Among the ligands meeting these criteria are derivatives of acridine [32], porphyrin [33], corrole [34] or carbazole [30]. Some of them, for example BRACO19 [35], pyridstatin [36], Phen-DC3 [37], L2H2-60TD [38] and L1H1-7OTD [39], are already commercially available and widely used in studies of G4s. However, the role of other structural G-quadruplex features cannot be ignored, as evidenced by other structurally different ligands interacting efficiently with G4s [27,40,41]. For example, the junction pocket between two G4 units may also serve as a target for specific recognition [42,43]. Moreover, dimeric G4 ligands target dimeric G4s, whereas tandemly aligned G4 ligands are able to discriminate a dimeric G4 from one that is monomeric as was shown for the dinickel salophen dimer [44], berberine dimer [45], and telomestatin derivative tetramer [46]. Nevertheless, in all cases, the challenge is to increase the affinity and specificity of such ligands, then G-quadruplex-targeting molecules would have the potential to become a very powerful new class of drugs. On the other hand, knowing that G-quadruplexes in proto-oncogenic or telomeric regions of the genome play significant roles in cancer progression, the classical antitumor drugs that interact with DNA have been tested to check their ability to bind with G4s [25]. For example, anticancer drugs, such as epirubicin [47] and adriamycin [48], bind as monomers to telomeric G4 with a high affinity. Therefore, in terms of the potential therapeutic application of G4s, recognizing ligands is necessary for them to be able to distinguish between different G4 topologies [49]. Of course, besides acting as potential therapeutic agents, ligands can be utilized as molecular agents in biosensing and bioimaging for diagnostic purposes [27,41]. A recent minireview by Asamisu et al. describes ligands’ design and development to acquire specificity and selectivity without compromising affinity [50]. For example, flat-shaped compounds (such as NMM IX or crystal violet) that were originally developed in different fields are often re- recognized as G-quadruplex binding ligands due to their planar geometry and availability [50].

**Figure 1:**
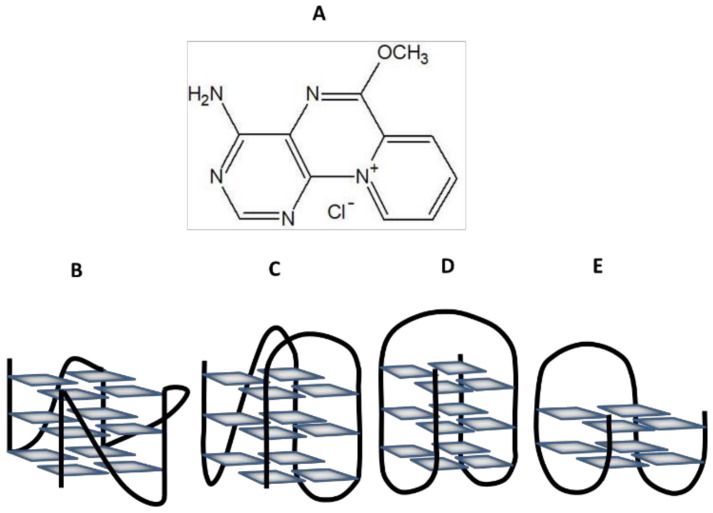
Structure of 9-methoxyluminarine chloride (**A**) and different G4s topology: parallel G4 (**B**), hybrid G4 (**C**), antiparallel chair-type G4 (**D**) and antiparallel basket-type G4 (**E**).

Thus, we present here studies of the interaction between 1-amino-9-methoxy-2,4,10-triaza-4b-azoniaphenanthrene (9-methoxyluminarine) and 9-MeLM with oligonucleotides, forming different structural G4s conformations (Figure 1). 9-MeLM is a small ligand with a heteroaromatic, flat structure, which also possesses a positive charge (Figure 1A). As 9-MeLM has no side-chains, we expected that all its interactions should be with a terminal G-quartet. Undeniably, the solubility, fluorescence, quantum yield, luminescence lifetime and photostability of 9-MeLM are also important factors that encouraged us to undertake investigations towards verifying its ability to serve as a selective G4s ligand.

9-Methoxyluminarine 9-MeLM is a cationic derivative of highly fluorescent nucleoside 7-(b’-D-ribofuranosylamino)-pyrido [2,1-h]pteridin-ll-ium-5-olate, termed luminarosine, bearing tricyclic betaine as an aglycone [51,52,53]. 9-MeLM also exhibits bright and stable fluorescence in the visible region as well as high quantum yield (Table 1). Among the halide ions, only I^−^ and, to a lesser extent, Br^−^ ions quench the fluorescence of 9-metoxyluminarine. So far, it has been shown that 9-MeLM showed moderate binding to calf thymus DNA with K_b_ value 2.8 × 10^4^ M^−1^ in 0–3.6 mM in the bp concentration range [53]. Taking into account the structural and photophysical properties of 9-methoxyluminarine, we assumed that 9-MeLM may indicate higher binding affinity to the G-quadruplexes than to double-stranded DNA. For our research, due to the structural diversity of G4s, we have chosen sequences that are able to form different G4 topologies, such as hybrid, antiparallel, and parallel G4 structures.

The human telomeric sequence d[AGGG(TTAGGG)_3_] (22HT) has been found to form different types of G-quadruplex structures. In K^+^ solution, two distinct (3 + 1) topologies—termed hybrid-1 and hybrid-2 G-quadruplex structures—were observed by NMR spectroscopy [54,55]. In an Na^+^ environment, an intramolecular antiparallel basket-type G-quadruplex structure with one diagonal and two lateral loops was observed by using the same technique [56]. Studying the interactions of ligands with G-quadruplex bearing the sequence of human telomeric DNA is very important in the context of research on cancer and telomerase, an active enzyme in cancer cells guaranteeing them an unlimited number of replications and “immortality” [57]. Antiparallel G-quadruplex, with the chair-type structure, is formed by thrombin-binding aptamer (TBA) in potassium ion solution [58,59,60]. This 15-mer oligonucleotide (5′-GGTTGGTGTGGTTGG-3′), with clotting activity at nanomolar concentration [61], adopts a unimolecular two-tetrad antiparallel G-quadruplex structure, composed of three lateral loops: two TT loops and a TGT loop protruding from the opposite sides of a two G-quartet moiety. Finally, we have chosen the parallel G-quadruplex as our molecular target. The intramolecular three-tetrad G-quadruplex, with three sidewise loops, is formed by the 5′-TGAGGGTGGGTAGGGTGGGTAA-3′ DNA sequence derived from the nuclease hypersensitive region of the *c-MYC* promoter (*c-MYC*) [4]. From the group of all proto-oncogenes, *c-MYC* is one of the most extensively studied and the best-characterized because of its involvement in cellular proliferation and cell growth, which may affect inhibition of the apoptosis [62,63].

Herein, we present the stability and interaction of 9-methoxyluminarine ligand 9-MeLM with G-quadruplexes formed by sequences corresponding to the human telomeric DNA, human proto-oncogene *c-MYC* and thrombin-binding aptamer.

## 2. Results

### 2.1. Fluorescence Studies

**Figure 2:**
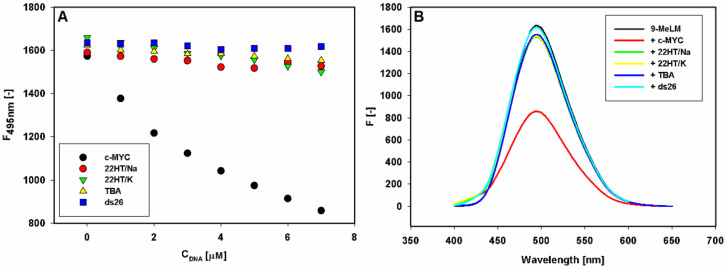
Fluorescence binding curves of 9-methoxyluminarine (9-MeLM) vs. the increasing concentration of G-quadruplexes (G4s) and double-stranded DNA (ds26) (**A**). Fluorescence spectra recorded for 9-methoxyluminarine in Tris-HCl buffer (10 mM, pH 7.2) w/o and with 7 equiv. of different G4s and ds26 (**B**).

First, 9-methoxyluminarine ligand (9-MeLM) in Tris-HCl buffer was excited at λ_ex_ = 390 nm and the recorded spectrum exhibited an unstructured emission band with a maximum of 495 nm. Next, fluorescence titration experiments were performed at a fixed concentration of 9-MeLM, by adding increasing amounts of each G-quadruplex or double-stranded DNA (ESI Figure S1). Importantly, the significant fluorescence quenching was observed only in the case of the c-*MYC* G-quadruplex (Figure 2).

In detail, 1 µM 9-methoxyluminarine solutions were titrated with 1 equiv. of pre-folded G4s solutions, and the corresponding fluorescence spectra, after each addition, were measured (ESI Figure S1A–D). Upon addition of double-stranded DNA (ds26), no changes in the fluorescence were observed up to ratio 7:1 ds26/9-methoxyluminarine (Figure 2).

The fluorescence quenching data were analyzed by the Stern–Volmer (SV) equation [64]:

F_0_/F = 1+ K_sv_ [Q] = 1 + K_q_ τ_0_ [Q], (1)

where F_0_ and F are the fluorescence intensities in the absence and presence of quenchers, respectively. K_q_, K_sv_, τ_0_ and [Q] are the quenching rate constant of the biomolecule, the Stern–Volmer quenching constant, the average lifetime of the molecule without the quencher and the concentration of the quencher, respectively.

**Figure 3:**
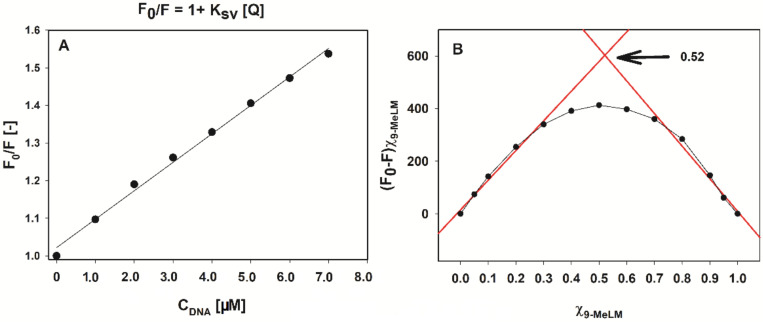
Fluorescence quenching Stern–Volmer plot of 9-methoxyluminarine (9-MeLM) with increasing concentration of c-*MYC* G-quadruplex (**A**). Job’s plot for determining the stoichiometry of the complex between 9-MeLM and G4 c-*MYC* (**B**).

A linear relationship of 9-methoxyluminarine fluorescence with c-*MYC* concentration was established as shown in Figure 3A (R^2^ = 0.99, concentration of c-*MYC* in the range of 0–7 µM with the slope of 7.56 × 10^4^ and intersect at 1.02). Therefore, we obtained a value of K_sv_ = 7.56 × 10^4^ M^−1^. Moreover, the formation of a non-fluorescent complex was confirmed by the quenching rate constants, K_q_, value, which were calculated using Equation (2):

K_q_ = K_sv_/τ_0,_ (2)

where the value of τ_0_ is the average molecule’s lifetime without the quencher. In our case, τ_0_ of 9-methoxyluminarine in water was estimated to be 10 ns (Table 1). Thus, the obtained K_q_ value is 7.56 × 10^13^ LM^−1^s^−1^ and it is much higher than the previously reported values for various quenchers in the presence of the biopolymer of 2 × 10^10^ LM^−1^s^−1^ (maximum value for scatter collision quenching constant) [64]. This result indicates that the quenching of 9-methoxyluminarine by c-*MYC* is a result of a complex formation and not induced by dynamic collision.

Moreover, we collected the fluorescence decays of 9-methoxyluminarine in the absence and presence of studied G-quadruplexes and double-stranded DNA. As an example, the experimental decays for 9-MeLM and 9-MeLM/c-*MYC* are shown in Figure S2. Each studied complex was prepared using ligand to DNA molar ratios of 1:10 as well as 1:5. In all cases, the mono-exponential fit was established with high goodness (χ^2^ < 1.2). The calculated fluorescence lifetimes are summarized in Table 2 and Table S1 for complexes 1:10 and 1:5, respectively. However, it can be seen that, when compared with free 9-methoxyluminarine, different topologies of DNA have not caused any significant changes in the 9-methoxyluminarine fluorescence lifetime. For the 9-MeLM/c-*MYC* complex in potassium we also measured fluorescence lifetimes for higher ratios of G-quadruplex versus 9-methoxyluminarine (15:1 and 20:1). In both cases, we observed negligible differences in lifetimes (9.88 ns vs. 9.90 ns, respectively). These results indicate the dominant role of the static quenching. Thus, we can assume that G-quadruplexes quench 9-methoxyluminarine fluorescence by static interactions.

For static quenching, the Stern–Volmer quenching constant (K_sv_) can be interpreted as the association constant or binding constant (K_b_) because static quenching comes from the formation of the complex between fluorophore and quencher. Therefore, we calculated K_sv_ for all tested G4 DNA samples and compared it with the binding constant (K_b_) calculated by the method given in the following section (Table 3).

### 2.2. Estimation of Binding Parameters

The fluorescence titration data were also quantitatively analyzed by constructing Benesi–Hildebrand (B-H) plots (Figure S3). We used the B–H method to estimate the binding affinities of the investigated ligand, because this method still allows data analysis though the binding plot does not reach saturation. The B–H model assumes that the stoichiometry of the formed ligand/G4 complex is 1:1 thus the result of the B–H calculation should be regarded as a nK_b_ product (Table 3) [65]. The differences between K_sv_ and K_b_ are not more than the order of magnitude. The calculated K_b_ is a few times higher for c-*MYC* in comparison with other G4s and the lowest binding constant was obtained between 9-MeLM and double stranded DNA. The K_b_ for 9-MeLM/ds26 (2.4 × 10^4^ M^−1^) is in good agreement with the K_b_ value (2.8 × 10^4^ M^−1^) estimated previously for 9-MeLM/CT-DNA using a Scatchard plot [53]. Additionally, the Job plot, based on the changes in the emission at 495 nm, was constructed for 9-MeLM/c-*MYC* (Figure 3B). The position of the maximum at X_9-MeLM_ = 0.52 confirmed the formation of a 1:1 stoichiometry of 9-MeLM/c-*MYC* complex. Moreover, it is known that the shape of the curve provides qualitative insight into K_eq_ and strong binding affords a more angular plot approaching the shape of a perfect triangle in the limit [66]. In our case, the shape of the curve (Figure 3B) is rather gentle, indicating a more balanced equilibrium. Importantly, the results obtained by the Benesi–Hildebrand method show a similar tendency as those obtained by the Stern–Volmer model (Table 3).

### 2.3. Circular Dichroism Studies

Circular dichroism spectroscopy (CD) is a very powerful technique for the determination of the structure, stability, and topology of G-quadruplexes, as well as the binding modes of ligands to nucleic acid structures [67,68,69,70]. We used CD spectroscopy to explain the effect of the 9-methoxyluminarine ligand 9-MeLM on the structure of four G4s with different conformations. This analytical technique can be used to effectively distinguish between hybrid, parallel and antiparallel G-quadruplex structures [70]. We also checked whether the G-quadruplex folding can be induced by a free ligand without the addition of sodium or potassium ions. At the same time, we used CD spectroscopy to determine the influence of the 9-MeLM ligand on the thermal stability of G4 DNA on the basis of the differences in the melting temperatures of the 3 equiv. of the ligand bound to G4 DNA and uncomplexed G4 DNA, as well as by analyzing the CD spectra recorded in the temperature range 10–90 °C under the same conditions.

Circular dichroism spectra of G-quadruplexes with different conformations are easily distinguishable. The typical CD spectrum of G-quadruplex with human telomeric sequence in the presence of K^+^ ion shows a strong positive peak at 293 nm with a distinctive shoulder around 270 nm and a smaller negative peak at 240 nm, which is characteristic of a mixed-type hybrid structure [71]. In the presence of Na^+^ ions, G-quadruplex adopts the antiparallel basket-type G-quadruplex structure with a characteristic CD spectrum showing a positive peak at 295 nm and two smaller peaks with the opposite orientation: a negative at 265 nm and a positive at 240 nm [56]. The same CD spectrum exhibits a 15-mer DNA sequence of a thrombin-binding aptamer, which forms a two-tetrad, antiparallel chair-type G-quadruplex structure in potassium ion solution [72]. The CD spectrum of a pre-annealed solution of G-quadruplex c-*MYC* in 100 mM KCl is dominated by a strong positive band at 260 nm and a smaller negative band at 240 nm, typical for the parallel G-quadruplex structure [70].

**Figure 4:**
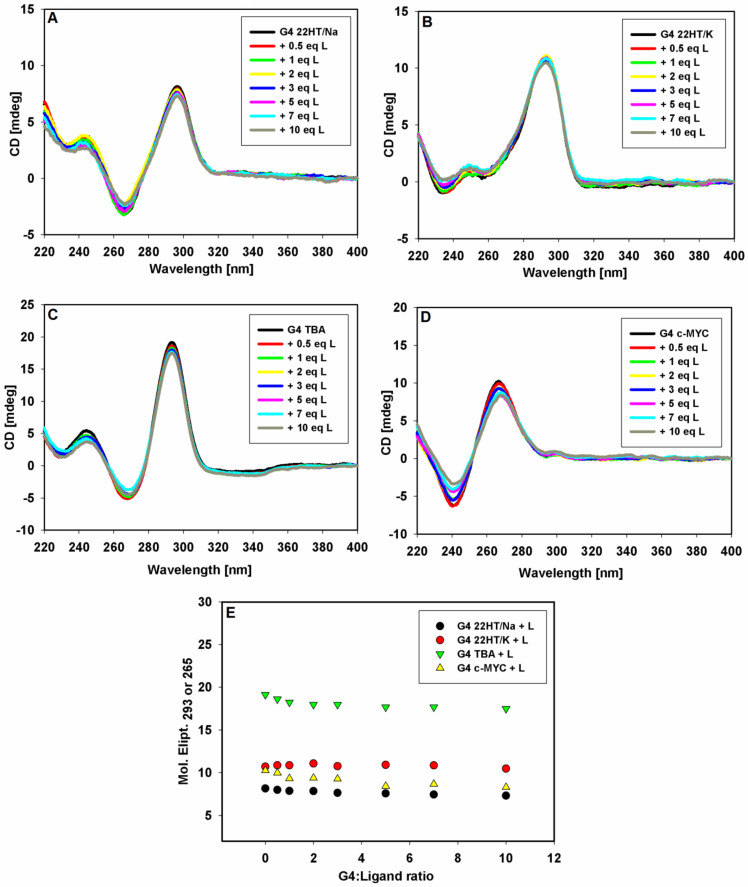
CD spectra of G-quadruplexes 22HT/Na (**A**), 22HT/K (**A**), TBA (**C**) and *c-MYC* (**D**) (5 μM) with increasing concentration of 9-MeLM ligand (L) in Tris-HCl buffer (10 mM, pH 7.2) containing 100 mM NaCl (**A**) or 100 mM KCl (**B**–**D**). Spectral changes at the selected wavelengths (293 and 265 nm) against G-quadruplexes DNA/ligand molar ratio (**E**).

The CD spectra of pre-annealed solutions of G-quadruplexes of different topologies in a buffer (10 mM Tris HCl, pH 7.2) containing 100 mM KCl or NaCl and were recorded (Figure 4). Upon the addition of each portion of 9-methoxyluminarine ligand, the sample was mixed and the CD spectrum was immediately performed. In general, the addition of the ligand did not affect the positions and intensity of the G4s CD signals, suggesting that the G-quadruplexes structures kept their topology. No significant changes in the CD spectra were observed even after the addition of ten equivalents of the ligand. Only in the case of G4 with a parallel structure did a slight decrease in the intensity of the CD signal suggest ligand-dependent perturbations in an ideal arrangement between tetrads in the c-*MYC* G-quadruplex [73]. Thus, the performed CD titration experiments of the G4s DNA with the ligand showed that 9-methoxyluminarine did not destabilize various G4 structures.

The induced signals (ICD) were not observed in the long-wavelength region where the achiral 9-methoxyluminarine ligand possesses an absorption band (λ_max_ = 390 nm) (Figure 4). Based on this observation, we can exclude the groove binding to G-quadruplex structures, because a positive ICD signal has been taken as an indicator of groove binding to G-quadruplex structures [67,69,74,75]. No changes observed in the long-wavelength range of CD spectra indicate that the structurally different G-quadruplexes, such as c-*MYC*, TBA and the telomeric ones, bind to the studied 9-methoxyluminarine ligand in the same way, probably by end-stacking interactions with external G-tetrads of G-quadruplexes.

**Figure 5:**
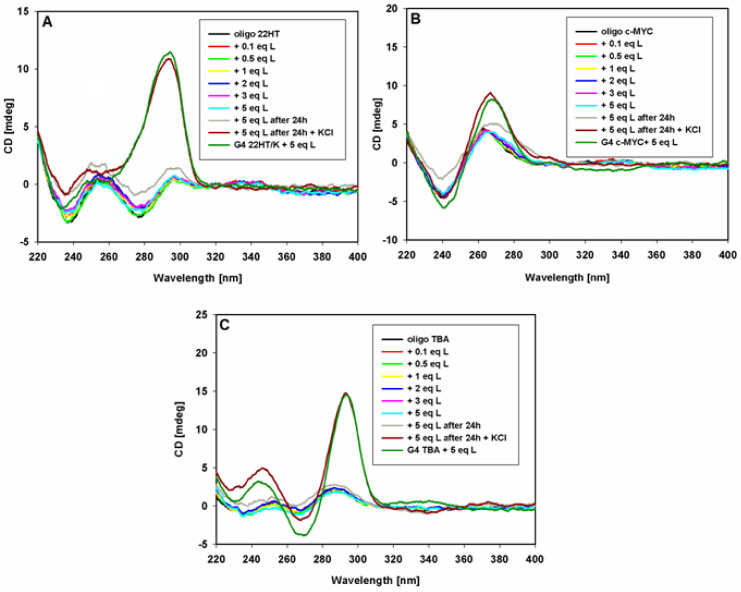
CD spectra of oligonucleotides 22HT (**A**), c-MYC (**B**) and TBA (**C**) (5 μM) with increasing amounts of 9-MeLM ligand (L) in Tris-HCl buffer (10 mM, pH 7.2).

The effect of 9-methoxyluminarine on telomeric, promoter c-*MYC* and thrombin-binding aptamer DNA oligonucleotides in the absence of added metal ions, as an inducer of G4, were also studied. The CD spectra in Tris HCl buffer at room temperature were recorded, and no significant changes were observed even after the addition of five equiv. of the ligand (Figure 5). Negligible changes were observed after 24 h. These results clearly show that for each tested sequence, G-quadruplex folding cannot be induced by a free ligand. As can be seen in Figure 5, KCl addition (100 mM) caused structural changes and the formation of the hybrid, parallel, and antiparallel G-quadruplex structures in the case of telomeric, c-*MYC* proto-oncogene and thrombin-binding aptamer sequences, respectively. These structures are fully formed G-quadruplexes, as evidenced by a comparison with the G4 spectra prepared the day before, to which five equiv. of ligand was added (Figure 5).

**Figure 6:**
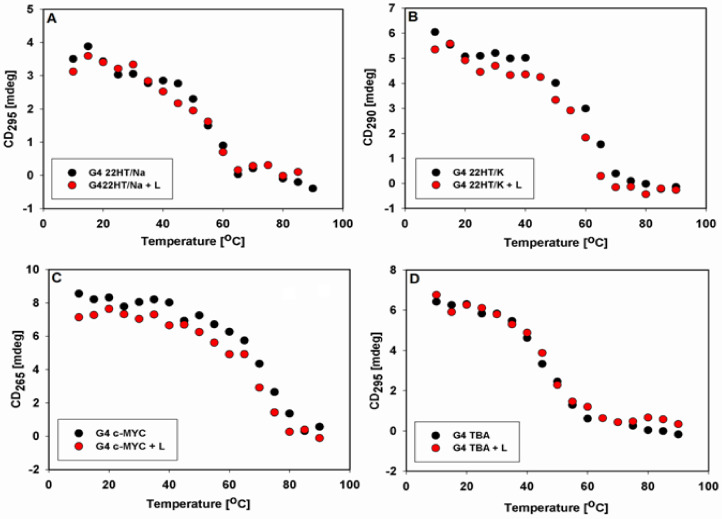
Intensity of CD signals of studied G4s without and in the presence of 9-MeLM (L) at 295 nm (**A**,**C**), 290 nm (**B**) and 265 nm (**D**) vs. temperature.

The influence of temperature on the CD spectra of the tested G-quadruplexes without and in the presence of 9-methoxyluminarine was also examined. CD spectra registered from 10 to 90 °C for all G-quadruplexes showed a gradual decrease in the intensity of signals characteristic of individual structures, which is consistent with the melting process (Figure S4). We constructed the curves at 295 nm (Figure 6A,D), 290 nm (Figure 6B) and 265 nm (Figure 6C) versus temperature for anti-parallel, hybrid and parallel topologies, respectively. The obtained sigmoidal curves indicated that G-quadruplexes are stable in the presence of 9-methoxyluminarine, 9-MeLM.

**Figure 7:**
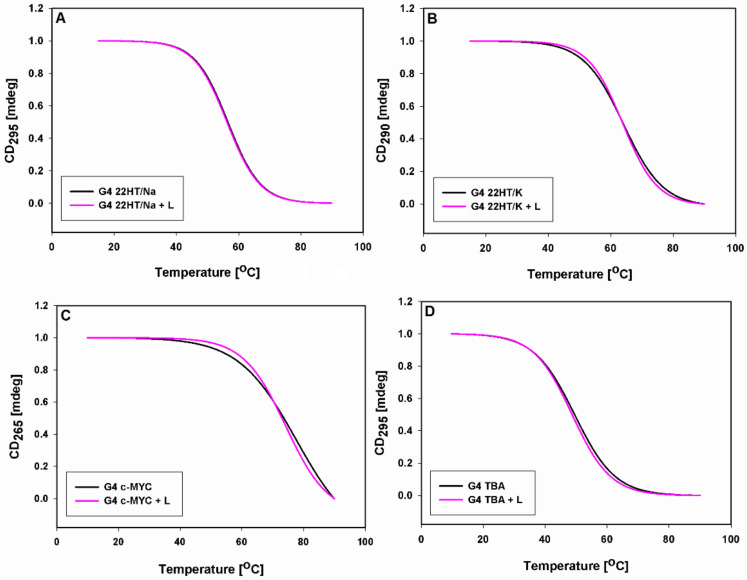
Normalized CD melting profiles (melting curves) of G-quadruplexes (2 µM) with and without 3 equiv. of 9-MeLM ligand (L) in 10 mM cacodylate buffer (pH 7.2) containing 100 mM NaCl (**A**), 100 mM KCl (**B**,**D**) and 10 mM KCl/90 mM LiCl (**C**).

The latter results were confirmed by CD melting study. The thermal denaturation profiles of G-quadruplexes DNA were monitored at wavelengths 295, 290 and 265 nm, at which there are the characteristic positive CD signals assigned to the typical antiparallel, hybrid and parallel G-quadruplex structures (Figure 7 and Figure S5). The melting curves were plotted in the absence and presence of 9-methoxyluminarine, which could show the thermal stabilization properties of ligand. The experiments were conducted in 10 mM cacodylate buffer (pH 7.2) containing 100 mM NaCl (22HT) or 100 mM KCl (22HT, TBA), as well as in buffer with low concentrations of stabilizing cation (K^+^ concentration reduced to 10 mM and the addition of 90 mM LiCl, c-*MYC*) [76]. 9-Methoxyluminarine shows no detectable stabilization of tested G-quadruplexes regardless of their structure (Figure 7, Table 4).

In all experiments, the melting and annealing processes proceeded without hysteresis indicating the same fast kinetics both with and without the 9-methoxyluminarine ligand (0.5 °C min^−1^ rates of temperature change). Only in the case of the 22HT G-quadruplex in sodium in the presence of 9-methoxyluminarine were the melting and annealing curves different from each other, which indicates a slightly slower kinetic process (Figure S5).

## 3. Discussion

To obtain information about the binding stoichiometry and constants for the complexes formed between 9-methoxyluminarine and G-quadruplexes, fluorescence experiments were carried out. Among all the DNA oligomers tested, 9-methoxyluminarine 9-MeLM exhibits a much higher preference for binding with G4-DNA, in particular with c-*MYC*. In order to explore the quenching mechanism, the fluorescence quenching process was firstly assumed to be dynamic quenching and the Stern–Volmer equation was constructed. The rate constant of the 9-methoxyluminarine quenching procedure initiated by c-*MYC* is greater than the K_q_ for various quenchers with biomolecules. This result means that quenching is not initiated by dynamic collision but by the formation of a non-fluorescent ground-state complex. The latter was further confirmed as no significant changes were observed in the lifetimes of free 9-MeLM in comparison with 9-MeLM/DNA complexes. Moreover, the mono-exponential decay for the bound 9-MeLM strongly implies the existence of one binding mode. The fluorescence Job’s plot also indicated 1:1 binding stoichiometry in the 9-MeLM/G4 *c-MYC* complex. Therefore, we assumed that the Stern–Volmer quenching constant (K_sv_) can be interpreted as a binding constant (K_b_). However, the binding constants obtained by the Stern–Volmer equation are a few times lower than those from the Benesi–Hildebrand estimations.

The results indicated that the binding affinities of 9-methoxyluminarine for the tested antiparallel and hybrid G4 DNA structures, as well as double-stranded DNA, were very comparable at the level of 10^4^ M^−1^, while the bond to the parallel G4 c-*MYC* structure was an order of magnitude stronger (Benesi–Hildebrand method). The binding mode between 9-methoxyluminarine and this G4 might involve a stronger π-π stacking interaction because of terminal G-tetrads unlimited by loops (three sidewise loops). The K^+^ stabilized human telomeric G4 DNA has the hybrid structure with two lateral loops and one sidewise loop. In the latter structure, one of the terminal G-tetrads is more accessible to the ligand than in the case of a sodium stabilized antiparallel basket-type human telomeric G-quadruplex structure (possessing one diagonal and two lateral loops) or a potassium stabilized antiparallel chair-type TBA G-quadruplex structure (with three lateral loops). Such binding affinities confirm all parameters determined using the Stern–Volmer model as well as the Benesi–Hildebrand method in the fluorescence titration experiments. Additionally, CD spectroscopy can provide some information on the binding mode of 9-MeLM to the four examined DNA conformations. When the tested 9-methoxyluminarine was added to G4s, no modification of the optical properties of the nucleic acids was observed. Stabilization of all examined G4 structures was noticed because no significant changes in the CD spectra were observed even after the addition of ten equivalents of the ligand. Only in the case of G4 with a parallel structure did a slight decrease in the intensity of the CD signal suggest ligand-dependent disorders in an ideal arrangement between tetrads in the c-*MYC* G-quadruplex. It should be emphasized that only this examined G4 structure has the sidewise-type of loops, which facilitate the ligand/G-tetrad π-π stacking interaction. The induced signals (ICD) were not observed in the long-wavelength region where the achiral 9-MeLM ligand possesses an absorption band (λ_max_ = 390 nm). Based on this observation, we can exclude the groove binding to G-quadruplex structures, because a positive ICD signal has been taken as an indicator of groove binding to G-quadruplex structures. On the basis of no changes observed in the long-wavelength range of the CD spectra, the binding process of 9-methoxyluminarine to the chosen structurally different G-quadruplexes DNA is proposed to occur via an end-stacking interaction with external G-tetrads.

## 4. Materials and Methods

### 4.1. Ligand

9-Methoxyluminarine, 9-MeLM was obtained as a gift sample from Prof. B. Skalski, Faculty of Chemistry, Adam Mickiewicz University, Poznań. The purity of the ligand was examined using the HPLC technique. The analysis was performed on an HPLC Agilent 1260 Infinity system, on X Terra MS C18 Column, (3.5 µm, 4.6 × 250 mm) at 25 °C, eluted with H20, using a linear gradient of 0–100% of acetonitrile over 15 min at a flow rate of 0.8 mL/min. The ligand sample was dissolved in H_2_O to obtain a stock solution of 1.3 mM concentration, which was stored at 4 °C.

### 4.2. Oligonucleotides

The quadruplex-forming 22-mer deoxyribonucleotides with a telomeric sequence of 5′-AGGG(TTAGGG)_3_-3′ (PDB ID: 143D and 2GKU), and a *c-MYC* sequence of 5′-TGAGGGTGGGTAGGGTGGGTAA-3′ (PDB ID: 2L7V), 15-mer with of anti-trombin aptamer TBA sequence 5′-GGTTGGTGTGGTTGG-3′ (PDB ID: 1RDE), as well as duplex DNA ds26 5′-CAATCGGATCGAATTCGATCCGATTG-3′, were purchased from Genomed (Warsaw, Poland) and were used without further purification. The strand concentrations were determined at 260 nm at 85 °C using extinction coefficients of 251,800 M^−1^cm^−1^ (22HT), 254,600 M^−1^cm^−1^ (*c-MYC*), 155,700 M^−1^cm^−1^ (TBA), 282,100 M^−1^cm^−1^ (ds26) as calculated from the published values of molar absorptivities of nucleotides [77].

Tris Base (CAS Number 77-86-1), Tris HCl (CAS Number 1185-53-1) and Dimethylarsinic acid sodium salt trihydrate (CAS Number 6131-99-3) were obtained from Aldrich Chemical Co. (Poznań, Poland) and used as received.

### 4.3. Steady State Fluorescence Measurements Fluorescence Spectroscopy

Fluorescence spectra were acquired using a Jasco Spectrofluorimeter (Tokyo, Japan). The emission spectra were recorded in the wavelength range of 400–750 nm at 25 °C for all the samples using an excitation wavelength (λ_ex_) of 390 nm. A quartz cuvette with a 10 mm path length in the excitation direction and a 4 mm path length in the emission direction were used and both excitation and emission slits were 5 nm.

### 4.4. Time Resolved Fluorescence Measurements

Fluorescence lifetimes were measured with a TimeHarp 200 PC-board for time-correlated single photon counting with 27 ps per channel resolution. The excitation source was a sub-nanosecond pulsed UV LED 370 with a maximum emission centered at 370 nm and a 0.75 ns wide pulse with full width at half maximum (FWHM), powered by a PDL 800-D driver. The emission was measured with a PMA 182 photon sensor head (all the instruments were from PicoQuant, Berlin, Germany).

The data were analyzed with an exponential reconvolution method using a non-linear least square fitting program. The time-resolved data were best fitted with a single exponential decay function. Optimum fitting with minimization of the residuals was confirmed using a Chi-squared value χ^2^ < 1.2. All fluorescence lifetimes measurements were performed at 22 °C, using the same quartz cuvette as for steady-state fluorescence spectra measurements.

### 4.5. Ligand-G4 Binding Study

Binding data obtained from spectrofluorimetric titrations were analyzed using the Benesi–Hildebrand transformation [65]. Experiments were carried out in the same manner—after each G4 DNA addition, the titrated solution was incubated for 3 min followed by the fluorescence spectrum measurement. The method of Benesi–Hildebrand, used to estimate the value of nK_b_, is represented by Equation (3), which describes the n-site ligand binding model:

1/F−F_0_ = 1/Fm−F_0_ + 1/(F_m_−F_0_)nK_b_ × 1/cG4 DNA, (3)

where F_0_ is the fluorescence of the ligand in the absence of G4 DNA, F is the fluorescence recorded in the presence of added G4 DNA, F_m_ is the fluorescence in the presence of added [G4 DNA]max, and *n* is the number of bound G-quadruplexes per ligand, and K_b_ is the binding constant.

The general procedure for drawing Job’s plot by the fluorescence method [78]: Stock solution of the same concentrations as those of 9-MeLM and *c-MYC* were prepared in the order of ≈4.0 × 10^−6^ ML^−1^ in Tris-HCl buffer (10 mM, pH 7.2) containing 100 mM KCl. The fluorescence in each case, with different 9-MeLM/*c-MYC* ratios but equal in volume (v = 1 mL), was recorded. Job’s plots were drawn by plotting ∆FX_9-MeLM_ vs. X_9-MeLM_ (∆F = change of fluorescence intensity of the spectrum and X_9-MeLM_ is the mole fraction of the 9-MeLM in each case, respectively).

### 4.6. Circular Dichroism

Circular dichroism (CD) spectra were recorded on a Jasco J-810 spectropolarimeter (Jasco, Tokyo, Japan), in the spectral range from 210 to 450 nm with a 200 nm/min scan speed and a bandwidth of 1 nm. Spectra were recorded in quartz cuvettes of 1 cm path length and were averaged from three scans. Measurements with G-quadruplexes and oligonucleotides were performed at 25 °C in a 10 mM Tris–HCl buffer (pH 7.2) containing 100 mM NaCl or KCl. Concentrations of DNA samples were 5 µM/strand. The ligand was added to G4 DNA and oligonucleotide solutions at increasing concentrations from 0.5 to 10 molar and 0.1 to 5 molar equivalents, respectively.

In the melting studies, the temperature of the samples was maintained by a Jasco Peltier temperature-controlled cell holder. Samples for melting profiles were prepared by mixing the 2 μM oligonucleotide solution in a 10 mM sodium cacodylate buffer (pH 7.2) and 100 mM NaCl (22HT/Na), 100 mM KCl (22HT/K, TBA), or 90 mM LiCl and 10 mM KCl (*c-MYC*) immediately before the experiment. The melting profiles were recorded in the absence and presence of 3 equiv. of ligand in 90–10 °C range with a 0.5 °C/min temperature gradient. All experiments were carried out using quartz cuvettes with a 10 mm optical path. Data were collected at 295 nm (22HT/Na, TBA), at 290 nm (22HT/K), and at 265 nm (*c-MYC*), using both cooling and heating approaches. Typically, two replicate experiments were performed, and the average values of the melting temperature are reported with a standard deviation of ±0.5 °C.

The effect of temperature was evaluated by heating the samples at 5 °C intervals from 10 to 90 °C and subsequently recording the CD spectrum after thermal equilibrium was attained. CD data (at 260, 290 or 295 nm) were collected in a temperature range of 10–90 °C in the presence of potassium or sodium ions.

## 5. Conclusions

We have shown that the fluorescence of the 9-metoxyluminarine ligand is selectively and effectively quenched by the *c-MYC* G-quadruplex. The quenching occurs via static mode, probably due to a π-π interaction between 9-MeLM and an exposed guanine tetrad of parallel G4. The 9-metoxyluminarine ligand is not able to induce G4s formation but also does not exhibit a destabilizing effect on G4s structures. Finally, we conclude that fluorescent measurements using 9-metoxyluminarine can provide a useful insight into the structure of G4s and should be a valuable tool in the preliminary assessment of G4s topology due to its ability to distinguish between parallel and other G4s structures.

## Figures and Tables

**Table 1:** Absorption and emission parameters for aqueous solution of 9-methoxyluminarine [53].

Absorption		Fluorescence		
^A^λ_max_ [nm]	ε [M^−1^ cm^−1^]	^F^λ_max_ [nm]	φ	τ[ns]
390	6800	489	0.99	10

**Table 2:** The fluorescence lifetimes calculated for 9-methoxyluminarine (9-MeLM) and its complexes with DNA (9-MeLM/DNA, 1:10) in Tris-HCl buffer (10 mM, pH 7.2). The estimated value of lifetime resolution is Δτ = 0.02 ns.

Cations	9-MeLM	9-MeLM/c-MYC	9-MeLM/22HT	9-MeLM/TBA	9-MeLM/ds26
100 mM K^+^	9.98 (ns)	9.86 (ns)	9.90 (ns)	10.00 (ns)	---
100 mM Na^+^	9.80 (ns)	---	9.90 (ns)	---	10.12 (ns)

**Table 3:** Parameters for the interaction of 9-methoxyluminarine (9-MeLM) with G-quadruplexes determined using S-V model as well as Benesi–Hildebrand method in fluorescence titration experiments (K_sv_—Stern–Volmer quenching constant, K_b_—binding constant, *n*—number of bound G-quadruplex molecules per ligand, λ_ex_ = 390 nm). ^c^ 10 mM KCl/90 mM LiCl.

DNA	Stern-Volmer Model, K_sv_ (×10^4^ M^−1^)	Benesi-Hildebrand Method, nK_b_ (×10^4^ M^−1^)
22HT/Na	0.8	4.8
22HT/K	1.9	6.2
c-*MYC* ^c^	7.6	19.0
TBA	0.6	3.9
ds26	0.4	2.4

**Table 4:** The melting temperatures T_m_ of G-quadruplexes (2 μM) free and incubated with 9-MeLM ligand (3 equiv.) in cacodylate buffer (10 mM, pH 7.2).

DNA	Tm [°C]	Tm + 9-MeLM [°C]
22HT/Na ^a^	56.3	56.1
22HT/K ^b^	63.0	63.7
c-*MYC* ^c^	71.5	71.3
TBA ^b^	48.5	48.1

^a,b,c^ T_m_ of G4s in the presence of ^a^ 100 mM NaCl; collected at 295 nm, ^b^ 100 mM KCl; collected at 290 and 295 nm and ^c^ 10 mM KCl/90 mM LiCl; collected at 265 nm (lit. 56 °C in 100 mM NaCl (22HT), 63.0 °C in 100 mM KCl (22HT), 50 °C (TBA) in a pH 7.0, 10 mM sodium cacodylic buffer [76]); lit. 75.5 °C, 20 mM KCl (c-*MYC*) [3]). Typically, two replicate experiments were performed, and average values are reported with a standard deviation of ±0.5 °C.

## Data Availability

All data are provided in the manuscript.

## Supplementary Materials

The following are available online at https://www.mdpi.com/article/10.3390/ijms221910399/s1, Figure S1: Fluorescence titration spectra of 9-methoxyluminarine (9-MeLM) ligand (1 µM) with G4 *c-MYC* (A), G4 TBA (B), G4 22HT/K (C) and G4 22HT/Na (D) (0–7 µM) in Tris-HCl buffer (10 mM, pH 7.2) containing 100 mM KCl (A–C) or 100 mM NaCl (D), Figure S2: Emission decay curves of 9-methoxyluminarine ligand w/o (A) and with G-4 c-MYC (at molar ratio 1:10) (B) in Tris-HCl buffer (10 mM, pH 7.2) containing 100 mM KCl, Table S1: The fluorescence lifetimes calculated for 9-methoxyluminarine and its complexes with DNA (9-MeLM/DNA, 1:5) in Tris-HCl buffer (10 mM, pH 7.2). The estimated value of lifetime resolution is Δτ = 0.02 ns, Figure S3: Benesi-Hildebrand plots of fluorescence binding data of 9-methoxyluminarine ligand with G-quadruplexes: *c-MYC* (A), 22HT/K (B), 22HT/Na (C), TBA (D), ds26 (E), Figure S4: CD spectra of G-quadruplexes: 22HT/Na (A,B), 22HT/K (C,D), *c-MYC* (E,F), TBA (G,H) (2 µM) in sodium cacodylate buffer (10 mM, pH 7.2) containing 100 mM NaCl (A, B) or 100 mM KCl C-H) recorded in the temperature range 10–90 °C. Spectra A, C, E, G registered in the absence of 9-methoxyluminarine ligand and B, D, F, H in the presence of ligand, Figure S5: Normalized CD melting profiles of G-quadruplexes (2 µM) with and without 3 equiv. of 9-MeLM (L) in 10 mM sodium cacodylate buffer (pH 7.2) containing 100 mM NaCl (A), 100 mM KCl (B,D) and 10 mM KCl/90 mM LiCl (C). The effect of heating and cooling on CD profiles of G4s in the absence and presence of 9-methoxyluminarine: solid lines are annealing curves, short dash lines are melting curves.
