# Supplementary material for: Interaction of 9-Methoxyluminarine with Different G-Quadruplex Topologies: Fluorescence and Circular Dichroism Studies
Source: Int J Mol Sci. 2021 Sep 27;22(19):10399. doi: 10.3390/ijms221910399 (PMC8508660; doi:10.3390/ijms221910399)
Supplement: Supplementary file 1 [file ijms-22-10399-s001.zip › ijms-1342411-supplementary.pdf]

Supporting Information

Interaction of luminarine with different G-quadruplex topologies: fluorescence and circular dichroism studies

Joanna Nowak-Karnowska[1], Agata Głuszyńska[1,*], Joanna Kosman[1], Grażyna Neunert[2], Anna Dembska[1,*]

[1] Department of Bioanalytical Chemistry, Faculty of Chemistry, Adam Mickiewicz University, Uniwersytetu Poznańskiego 8, Poznań 61-614, Poland;

[2] Department of Physics and Biophysics, Faculty of Food Science and Nutrition, Poznań University of Life Sciences, Wojska Polskiego 38/42, 60-637 Poznan, Poland

[*] Correspondence: aglusz@amu.edu.pl (A.G.), aniojka@amu.edu.pl (A.D.)

Contents:

Figure S1. Fluorescence titration spectra of 9-methoxyluminarine (9-MeLM) ligand (1 μM) with G4 *c-MYC* (A), G4 TBA (B), G4 22HT/K (C) and G4 22HT/Na (D) (0–7 μM) in Tris-HCl buffer (10 mM, pH 7.2) containing 100 mM KCl (A-C) or 100 mM NaCl (D).

Figure S2. Emission decay curves of 9-methoxyluminarine ligand w/o (A) and with G-4 *c-MYC* (at molar ratio 1:10) (B) in Tris-HCl buffer (10 mM, pH 7.2) containing 100 mM KCl.

Table S1. The fluorescence lifetimes calculated for 9-methoxyluminarine and its complexes with DNA (9-MeLM/DNA, 1:5) in Tris-HCl buffer (10 mM, pH 7.2). The estimated value of lifetime resolution is Δτ = 0.02 ns.

Figure S3. Benesi-Hildebrand plots of fluorescence binding data of 9-methoxyluminarine ligand with G-quadruplexes: *c-MYC* (A), 22HT/K (B), 22HT/Na (C), TBA (D), ds26 (E).

Figure S4. CD spectra of G-quadruplexes: 22HT/Na (A, B), 22HT/K (C, D), c-*MYC* (E,F), TBA (G,H) (2 μM) in sodium cacodylate buffer (10 mM, pH 7.2) containing 100 mM NaCl (A,B) or 100 mM KCl C-H) recorded in the temperature range 10-90 °C. Spectra A, C, E, G registered in the absence of 9-methoxyluminarine ligand and B, D, F, H in the presence of ligand.

Figure S5. Normalized CD melting profiles of G-quadruplexes (2μM) with and without 3 equiv. of 9-MeLM (L) in 10 mM sodium cacodylate buffer (pH 7.2) containing 100 mM NaCl (A), 100 mM KCl (B, D) and 10 mM KCl/90 mM LiCl (C). The effect of heating and cooling on CD profiles of G4s in

the absence and presence of 9-MeLM : solid lines are annealing curves, short dash lines are melting curves.

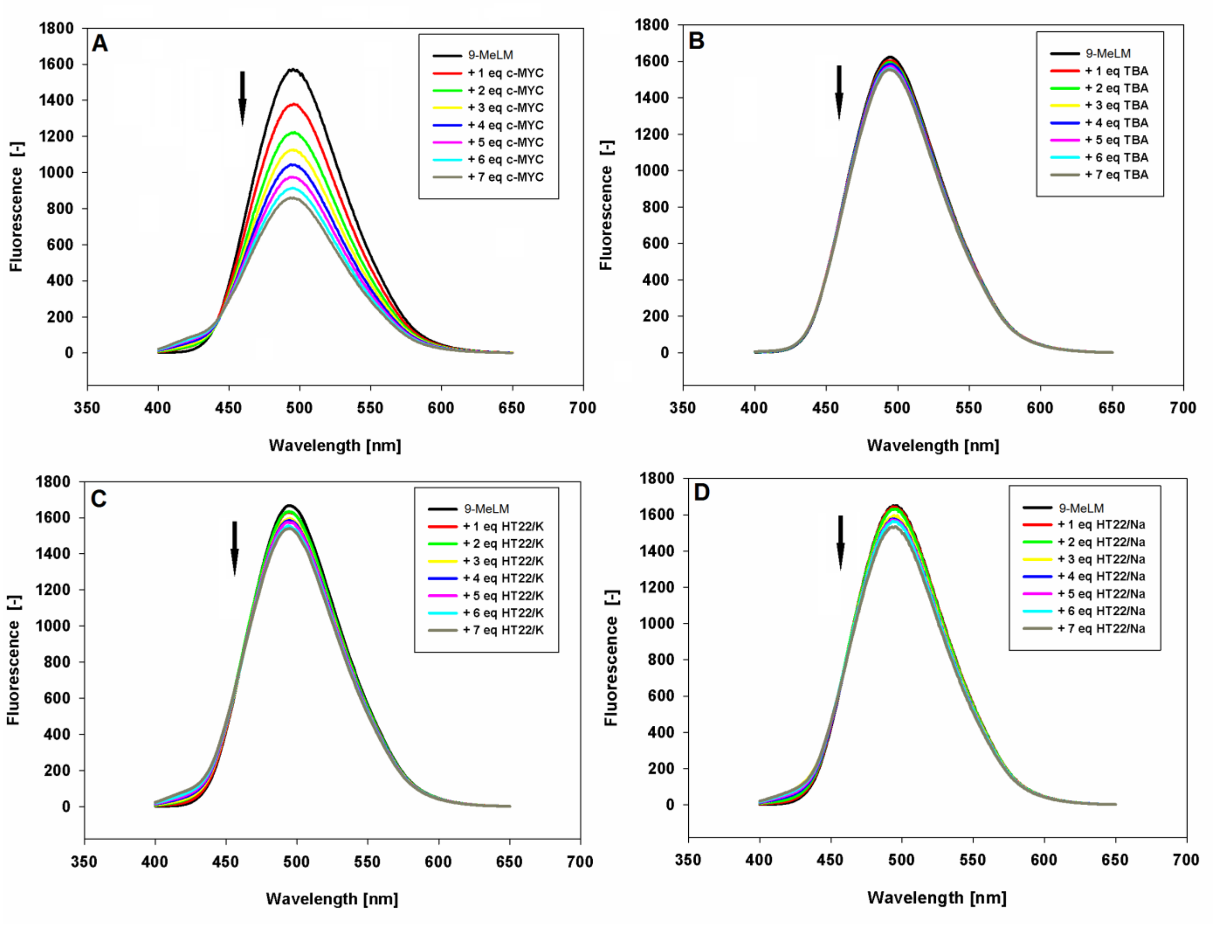

Figure S1. Fluorescence titration spectra of 9-methoxyluminarine (9-MeLM) ligand (1 µM) with G4 *c-MYC* (A), G4 TBA (B), G4 22HT/K (C) and G4 22HT/Na (D) (0–7 µM) in Tris-HCl buffer (10 mM, pH 7.2) containing 100 mM KCl (A-C) or 100 mM NaCl (D).

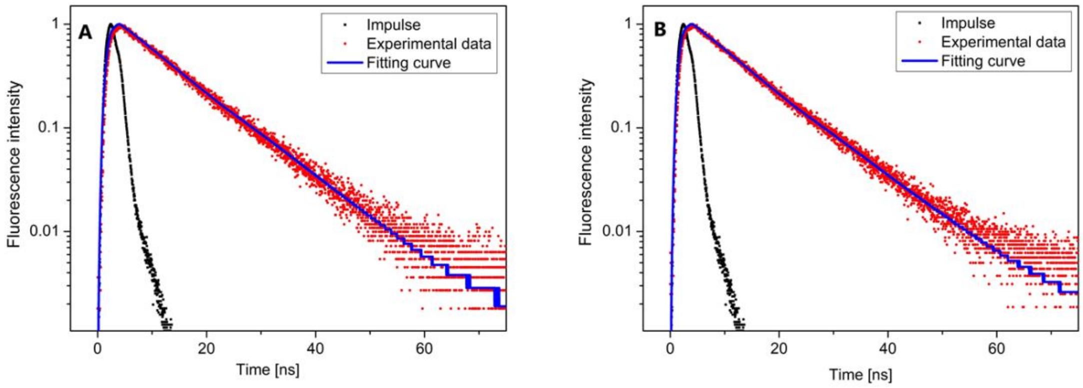

Figure S2. Emission decay curves of 9-methoxyluminarine ligand w/o (A) and with G-4 *c-MYC* (at molar ratio 1:10) (B) in Tris-HCl buffer (10 mM, pH 7.2) containing 100 mM KCl.

Table S1. The fluorescence lifetimes calculated for 9-methoxyluminarine and its complexes with DNA (9-MeLM/DNA, 1:5) in Tris-HCl buffer (10 mM, pH 7.2). The estimated value of lifetime resolution is $\Delta\tau = 0.02$ ns.

**Cations**	**9-MeLM**	**9-MeLM/*c-MYC***	**9-MeLM/22HT**	**9-MeLM/TBA**	**9-MeLM/ds26**
100 mM K^+	9.98 (ns)	9.98 (ns)	9.96 (ns)	9.96 (ns)	---
100 mM Na^+	9.80 (ns)	---	9.96 (ns)	---	10.10 (ns)

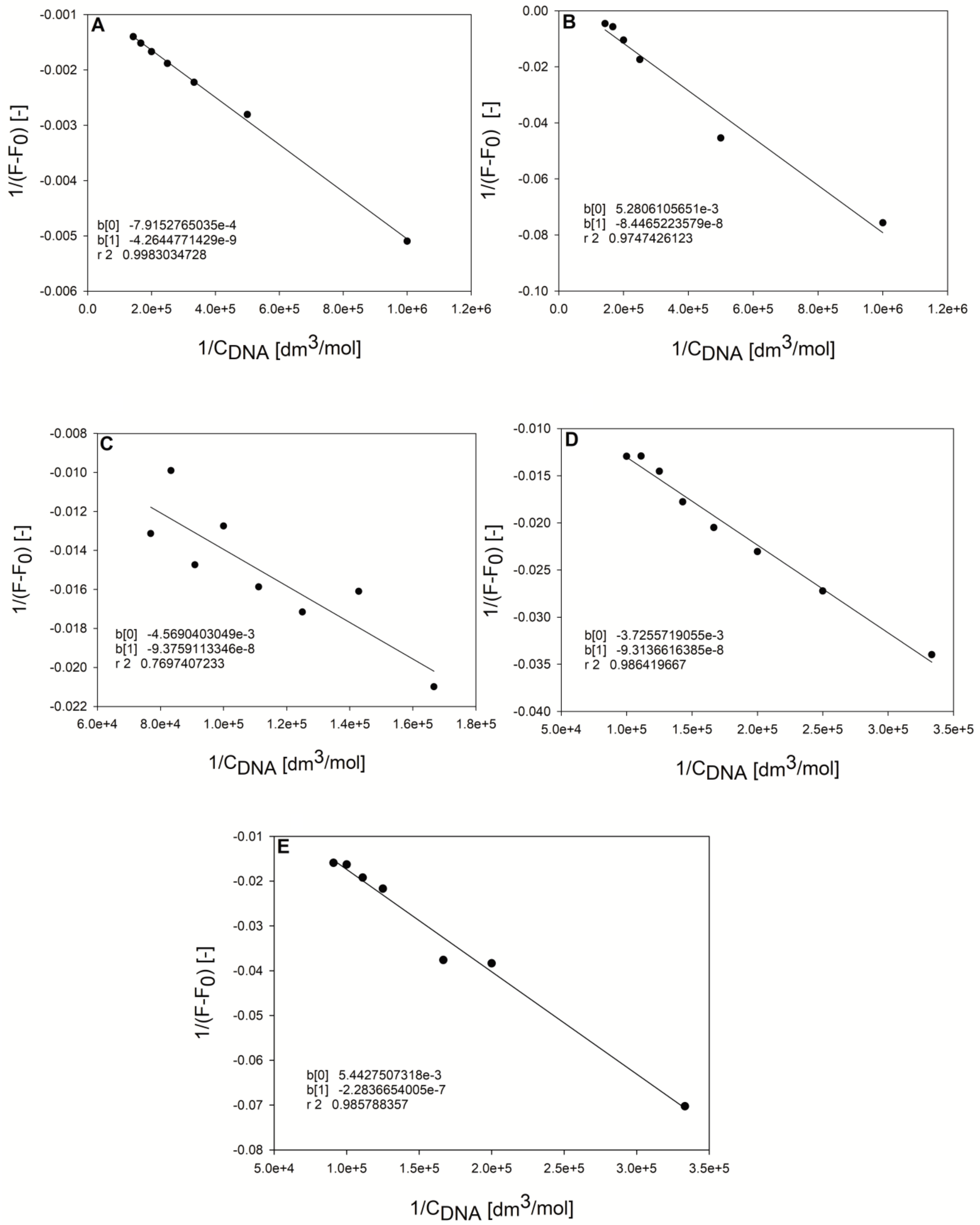

Figure S3. Benesi-Hildebrand plots of fluorescence binding data of 9-methoxyluminarine ligand with G-quadruplexes: *c-MYC* (A), 22HT/K (B), 22HT/Na (C), TBA (D), ds26 (E).

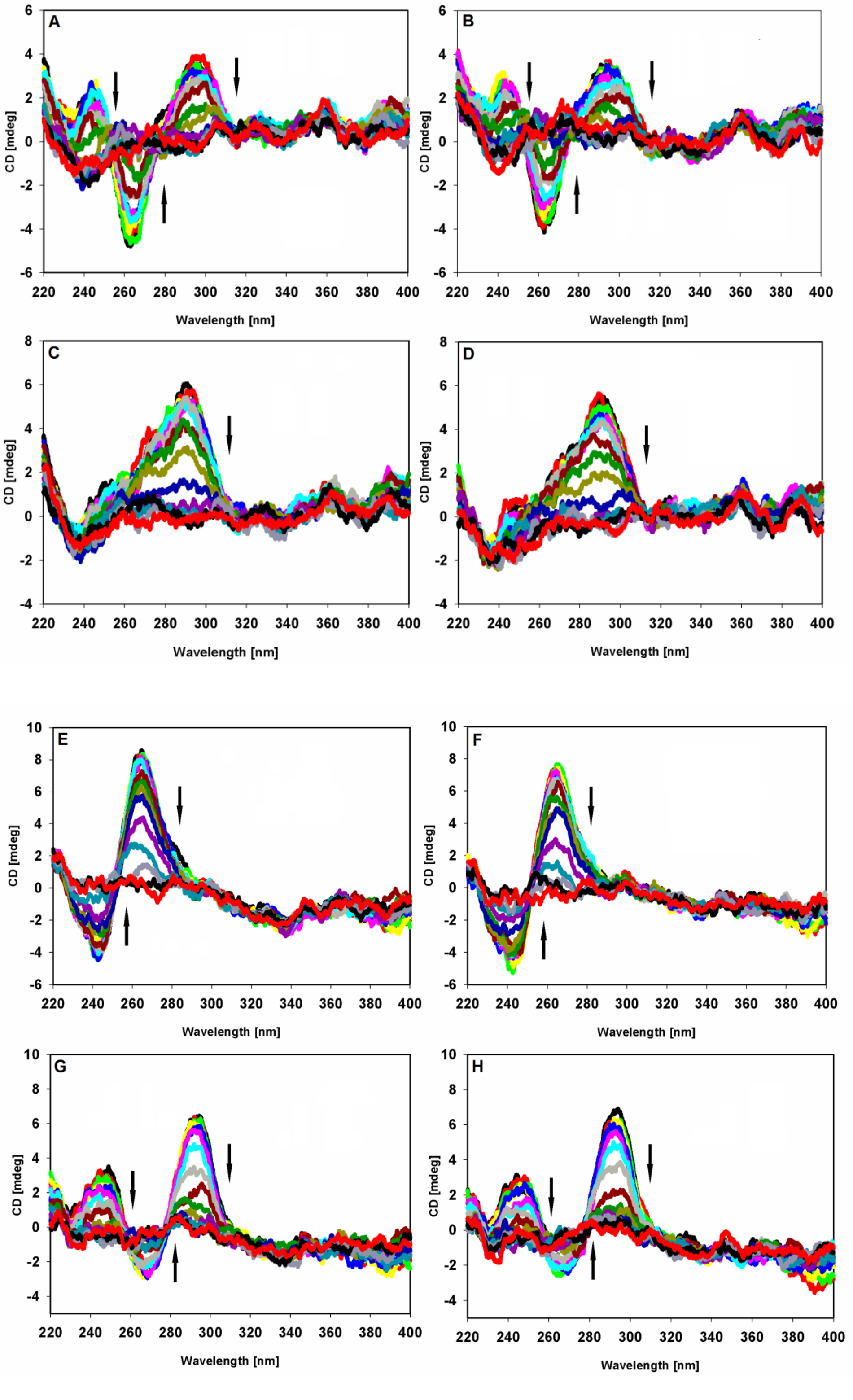

Figure S4. CD spectra of G-quadruplexes: 22HT/Na (A, B), 22HT/K (C, D), c-*MYC* (E,F), TBA (G,H) (2 μM) in sodium cacodylate buffer (10 mM, pH 7.2) containing 100 mM NaCl (A,B) or 100 mM KCl C-H) recorded in the temperature range 10-90 °C. Spectra A, C, E, G registered in the absence of 9-methoxyluminarine ligand and B, D, F, H in the presence of ligand.

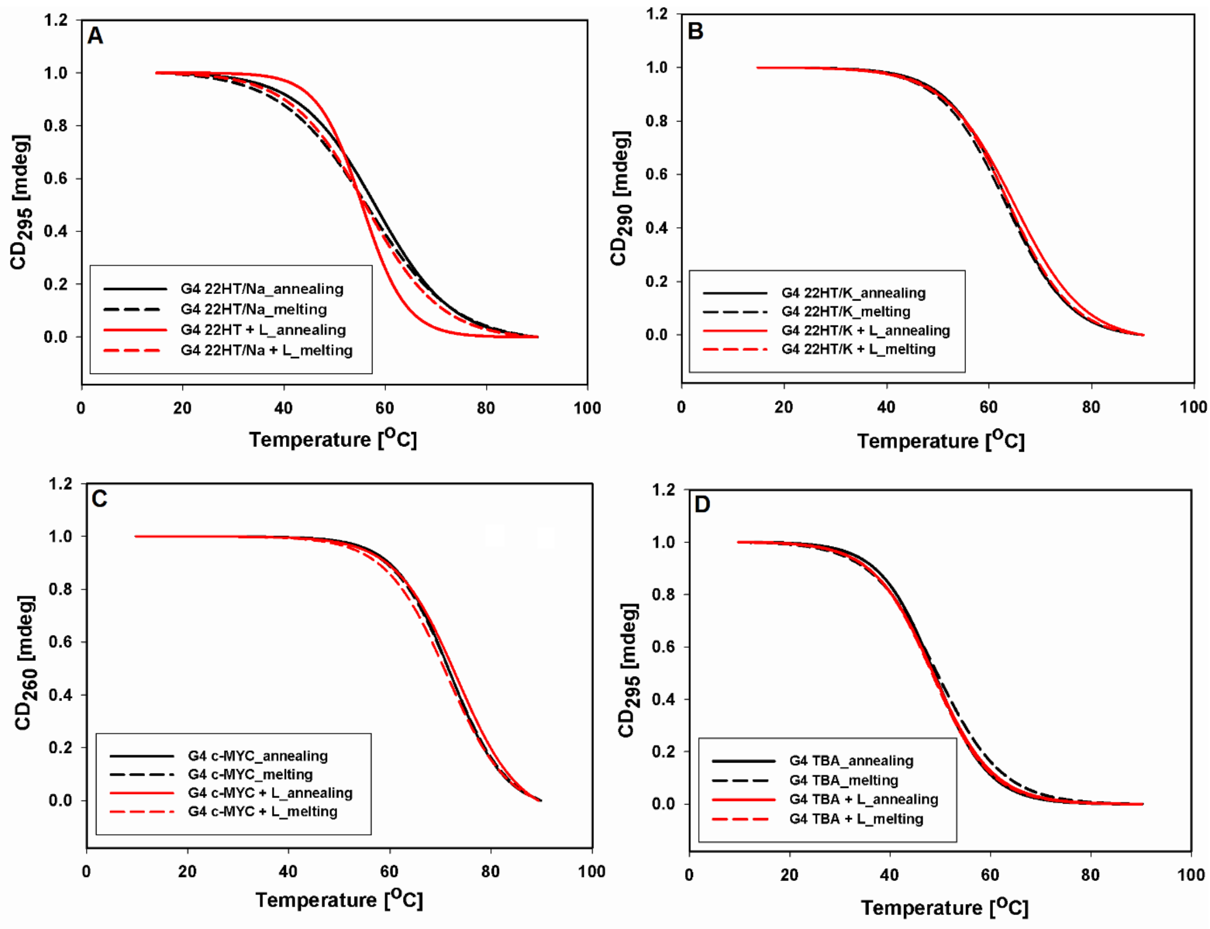

Figure S5. Normalized CD melting profiles of G-quadruplexes (2µM) with and without 3 equiv. of 9-MeLM (L) in 10 mM sodium cacodylate buffer (pH 7.2) containing 100 mM NaCl (A), 100 mM KCl (B, D) and 10 mM KCl/90 mM LiCl (C). The effect of heating and cooling on CD profiles of G4s in the absence and presence of 9-MeLM: solid lines are annealing curves, short dash lines are melting curves.
